# Protocol for a randomised controlled trial of a web-based healthy relationship tool and safety decision aid for women experiencing domestic violence (I-DECIDE)

**DOI:** 10.1186/s12889-015-2072-z

**Published:** 2015-08-01

**Authors:** Kelsey Hegarty, Laura Tarzia, Elizabeth Murray, Jodie Valpied, Cathy Humphreys, Angela Taft, Lisa Gold, Nancy Glass

**Affiliations:** Department of General Practice, The University of Melbourne, Melbourne, Australia; Department of Social Work, The University of Melbourne, Melbourne, VIC Australia; Judith Lumley Centre, La Trobe University, Melbourne, VIC Australia; eHealth Unit, Department of Primary Care & Population Health, University College, London, UK; School of Nursing, Johns Hopkins University, Baltimore, MD USA; Department of Population Health, Deakin University, Melbourne, VIC Australia

**Keywords:** Domestic violence, Randomised controlled trial, Protocol, e-Health, Web-based tools, Safety, Internet, Women, Technology, Self-help

## Abstract

**Background:**

Domestic violence is a serious problem affecting the health and wellbeing of women globally. Interventions in health care settings have primarily focused on screening and referral, however, women often may not disclose abuse to health practitioners. The internet offers a confidential space in which women can assess the health of their relationships and make a plan for safety and wellbeing for themselves and their children. This randomised controlled trial is testing the effectiveness of a web-based healthy relationship tool and safety decision aid (I-DECIDE). Based broadly on the IRIS trial in the United States, it has been adapted for the Australian context where it is conducted entirely online and uses the Psychosocial Readiness Model as the basis for the intervention.

**Methods/design:**

In this two arm, pragmatic randomised controlled trial, women who have experienced abuse or fear of a partner in the previous 6 months will be computer randomised to receive either the I-DECIDE website or a comparator website (basic relationship and safety advice). The intervention includes self-directed reflection exercises on their relationship, danger level, priority setting, and results in an individualised, tailored action plan. Primary self-reported outcomes are: self-efficacy (General Self-Efficacy Scale) immediately after completion, 6 and 12 months post-baseline; and depressive symptoms (Centre for Epidemiologic Studies Depression Scale, Revised, 6 and 12 months post-baseline). Secondary outcomes include mean number of helpful actions for safety and wellbeing, mean level of fear of partner and cost-effectiveness.

**Discussion:**

This fully-automated trial will evaluate a web-based self-information, self-reflection and self-management tool for domestic violence. We hypothesise that the improvement in self-efficacy and mental health will be mediated by increased perceived support and awareness encouraging positive change. If shown to be effective, I-DECIDE could be easily incorporated into the community sector and health care settings, providing an alternative to formal services for women not ready or able to acknowledge abuse and access specialised services.

**Trial registration:**

Trial registered on 15^th^ December 2014 with the Australian New Zealand Clinical Trials Registry ACTRN12614001306606

## Background

Domestic violence (DV) is prevalent worldwide, and is linked to a range of negative health effects and outcomes for women [1]. It is defined as any behaviour within an intimate relationship that causes physical, psychological or sexual harm to those in the relationship [2], and can include emotional abuse and threats, stalking, pushing, hitting, punching, assault with weapons, and forced sex. The case for addressing this devastating social problem and its associated health impacts as a matter of urgency has been made repeatedly, yet, to date, there is little evidence to support effective interventions [3–8]. A challenge for any intervention is the need to address women’s varied experiences of violence and their individual circumstances and readiness for action [9, 10]. Furthermore, concerns around privacy, confidentiality, and safety may present barriers to disclosure, identification, and uptake of referrals to formal services [8].

### Evidence informing the development and design of I-DECIDE

A recent primary care study, *weave* [11], attempted to overcome these barriers through a multi-faceted intervention with women fearful of a partner in the previous 12 months offered brief counselling from specially-trained family doctors. Doctors in the intervention group enquired more about safety of women and children, and the women reported less depressive symptoms and higher self-efficacy than the comparison group receiving usual care, with no change in primary outcomes [11]. However, only half of the women took up the invitation for *weave* counselling. Barriers included not seeing doctors as an avenue to help because of doctors’ prior poor communication or being seen as providers of physical health care only with limited time available, and women also described the difficult issue of managing disclosure face to face [12]. Other studies have found similar barriers of lack of access to quality care, worries about confidentiality and judgemental responses by face to face interventions, [13] with women appearing to prefer disclosing abuse online to face-to-face [14]. Further, personal delivery of safety planning requires highly trained staff, who are not generally widely available and health practitioners are often reluctant to undertake this work [15]. Thus, relatively few women have the opportunity to assess their own danger level or to systematically weigh up competing factors for safety. These factors include maintaining privacy, protecting children, feelings for their partner, history and severity of violence, likelihood of escalation if they leave, and access to support and resources [16].

The delivery of interventions online has the potential to overcome these barriers, as they can be accessed anonymously, at a time and place of the woman’s choosing, without the need to disclose the violence to a professional. The internet is being increasingly harnessed as a method of delivery for interventions to address sensitive, stigmatising conditions [17], including mental health issues [18] and sexual health. To date, little research has explored the possibility of utilising the internet for DV responses. One study evaluating the effectiveness of a web-based intervention for DV is the IRIS trial (NCT01312103) in the United States [19]. The IRIS intervention is informed by Dutton’s empowerment model [20] and focuses on reducing women’s decisional conflict and increasing safety planning and behaviours. The safety process involves complex individual, cultural and community factors, and IRIS is designed to help women experiencing abuse to identify safety priorities and develop a personalised safety plan while considering staying or leaving an abusive relationship. Preliminary work suggests that women felt more supported and had less decisional conflict after only a single use [19]. Subsequent related international work conducted in New Zealand (ACTRN12612000708853) [21] and in Canada (NCT02258841) is evaluating similar safety decision online interventions to promote safety and improved mental health. In Australia, the I-DECIDE About my Relationship website builds on and acknowledges this work, with the addition of a healthy relationship tool to the safety decision aid. The emphasis is on helping women to self-inform, self-reflect, and self-manage on their pathway to safety and healing for themselves and their children. I-DECIDE incorporates brief counselling techniques such as motivational interviewing to increase awareness and non-directive problem solving to assist with taking action [22].

### Aims of I-DECIDE

The primary aim of the I-DECIDE trial is to determine if an interactive web-based healthy relationship tool and safety decision aid compared to a non-interactive information only website results primarily in:

- Increased self-efficacy
- Decreased depressive symptoms

The secondary aims are to determine if the intervention results in:

- Increased helpful actions for safety and well being
- Decreased fear of their partner and is cost effective.

The intervention website consists of self-reflection on behaviours from their partner, a danger assessment, messaging about their relationship and their safety, weighing up different priorities for their relationship and a tailored action plan). We hypothesise that the intervention will increase women’s perceived support and awareness of the abuse, which will increase their readiness for action with regard to their relationship and their self-efficacy and that these internal changes will lead to increases in safety and wellbeing actions and improvement in women’s depressive symptoms (see Tables 1, 2 and 3).

**Table 1:** Primary outcome measures

Outcome	Measure	Description & purpose	Hypothesis
Self-efficacy	The General Self-Efficacy Scale	The scale assesses a general sense of perceived self-efficacy, aiming to predict coping ability and adaptation to stressful life events. The Scale has 10 questions with response choices on a 4-point scale: Not at all true/Hardly true/ Moderately true /Exactly true.	A higher mean self-efficacy score than the comparison group, by at least a third of a standard deviation, as measured by the General Self-Efficacy Scale [34], immediately after intervention completion and at 6 and 12 months post-baseline.
Depression	The Center for Epidemiologic Studies Depression Scale, Revised (CESD-R)	The Center for Epidemiologic Studies Depression Scale (CESD) is widely used in community samples. The 20 items in the CESD-R scale measure symptoms of depression in nine different groups as defined by the DSM-IV. Users are asked to rate the occurrence of symptoms ‘in the past week or so’ from 0 (Rarely or none of the time – less than one day) up to 4 (Nearly every day for two weeks)	A lower mean depression score than the comparison group, by at least a third of a standard deviation, as measured by The Center for Epidemiologic Studies Depression Scale, Revised (CESD-R) [35] at 6 and 12 months post-baseline.

**Table 2:** Secondary outcome measures

Outcome	Measure	Description & purpose	Hypothesis
Actions for safety and wellbeing	weave service use & activities questionnaire (modified)^a^	These questions drawn from the *weave* study [11] ask women what activities or services she has used over the past 6 months. If she answers ‘yes’ to any items, a second question drops down asking whether it was helpful (yes/no).	A higher mean number of actions for safety and wellbeing that are helpful than the comparison group, at 6 months post-baseline.
	Self-care activities questionnaire^1^	These questions are a modified list of self-care activities derived from the *Diamond* study on depression [36]. Women are asked if they have started doing, or increased the frequency, of any of the listed self-care activities. If she answers ‘yes’ to any items, a second question drops down asking whether it was helpful (yes/no).	
Fear of partner	Visual Analogue scale 0-100	The participant will be asked to rate their current level of fear of their partner or ex-partner, on a sliding scale from 0 (not at all afraid) to 10 (very afraid).	A lower mean fearfulness score than the comparison group, as measured on a visual analogue scale of current level of fear of partner (0–100), at 12 months post-baseline

^a^These questions were developed for the purposes of this study

**Table 3:** Mediators of the outcomes

Measure	Outcome	Description & purpose
Perceived support - visual analogue scale^a^	Perceived support (website)	The participant will be asked to rate how supported they feel by the website on a sliding scale from 0 (completely unsupported) to 10 (completely supported).
Contemplation Ladder, modified version^a^	Awareness	Women will indicate their position on a modified version of the Contemplation Ladder [37], a tool originally developed to measure readiness to cease smoking. The ladder is designed to measure awareness of abuse from 0–10 based on how ready the woman is to make positive changes to her situation.

^a^These questions were developed for the purposes of this study

**Table 4:** Moderators of the outcomes

Measure	Outcome	Description & purpose
*Diamond* life event questionnaire	External events	This questionnaire was taken from the *Diamond* longitudinal depression study [36]. Questions ask whether a woman has experienced any of 13 significant life events over the last 12 months, and if so, whether it had a positive or negative impact.
Medical Outcomes Survey - Social Support, 5-item version (MOS-SS5)	Perceived support (social)	This is a 5-item version of the MOS social support survey. The questions ask the woman how often she has access to support from someone in her life, with response options on a 5-point Likert scale.

## Methods/design

The I-DECIDE trial design is a two-arm pragmatic randomised controlled trial to test the effectiveness of a web-based healthy relationship tool and safety decision aid. The protocol is described according to CONSORT-EHEALTH guidelines [23]. Ethics approval for this study was obtained by the Human Ethics Research Committee at the University of Melbourne (HREC 1442953).

### Participant inclusion criteria

The target population for the I-DECIDE study is English-speaking women aged 16–50 years residing in Australia who are, or have been, in an abusive relationship or experienced fear of an (ex) partner in the past 6 months. This is determined by a positive response to one or more of the following: in the past 6 months a partner or ex-partner has made her feel afraid or unsafe; has followed her or harassed her over the telephone or online; has called her names, humiliated, bullied or criticized her, or threatened her in any way; has isolated her from her family and friends or restricted her behaviour in any way; or has physically harmed her in any way; or has forced her to do sexual things she didn’t want to. Additionally, eligible women need to have access to a safe computer and internet connection, be willing to provide their full name, current postal address, and a valid email address, as well as first name, email or phone number for at least one safe contact person. Women are excluded if in a follow up phone call they identify that they have not been in an unhealthy or abusive relationship or experienced fear of partner in the past 6 months.

### Number of participants required

A final sample size of at least 141 women in each of the two groups will be required to detect differences of a third of a standard deviation between group means on the primary outcomes (see Table 1) at 6 and 12 months, with at least 80 % power (alpha 5 %, 2-sided test). Attrition of up to 30 % has been found in previous studies with this population in Australia [11] and the US [24]. Allowing for an attrition rate of 30 % by the 12 month follow-up time point, at least 404 eligible women will need to be recruited to the baseline phase of the trial (202 women in each group).

### Recruitment

**Fig 1:**
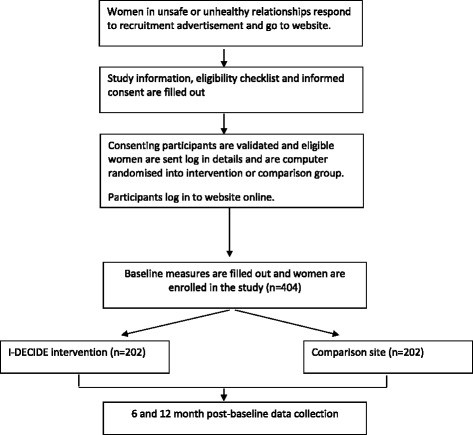
Flow of participants through trial

Recruitment for the I-DECIDE study is being conducted entirely online (see Fig. 1). This allows the study to more closely mimic the way that I-DECIDE might be used in a real-life setting, and thus provide a more realistic assessment of its effectiveness. A mixture of targeted women’s health or domestic violence-related websites, social media (e.g. Facebook), and online advertising are being utilised. These sources can provide access to a wide range of women, not only those who have already acknowledged the violence and are using support services. Interested women click directly on the link provided in the advertisement which directs them to the I-DECIDE homepage (www.idecide.org.au). A toll-free phone number is provided for women to contact the research team with queries, as well as a study email address for technical difficulties.

### Trial process

From the I-DECIDE homepage (www.idecide.org.au) women must click ‘Be a part of the project’ to participate. This directs them to the eligibility screening questionnaire, participant information statement, and consent form. A woman is considered eligible once she has met eligibility criteria, provided informed consent, and provided all the requested contact details. Once she clicks ‘Submit’, the woman is randomised by computer algorithm into either the intervention or comparison arm, and receives a standard automated email with the corresponding URL and password. She then has a 12 week window in which to log in to complete her baseline visit and be enrolled. Women are unable to determine their allocation until after they fill out the baseline measures and proceed to the intervention or comparison tool. Automated reminder emails are sent at regular intervals throughout the 12 week window until the woman completes baseline. To minimise the risk of fraudulent participants, women’s details are validated manually by a research assistant against the Australian Electoral Roll. If their details do not match any on the Electoral Roll, they are contacted by email asking for further details in order to be able to complete validation. If the participant is still not able to be validated, she is deemed ineligible and her data are removed.

Completion of the baseline study measures is estimated to take up to 20 min. For women who then proceed to the intervention, the overall time commitment could be up to 60 min, and for the comparison group up to 40 min. Women are not required to complete their visit all in one sitting, but can log out and back in at another time.

For 6 and 12 month follow-up, automated reminder emails will alert women that they are due to complete another session. Women will be reminded of their username and password, and the URL. When women log back in to the website, the questions will have changed over to the 6 or 12 month outcome assessments. Women in the intervention group may choose to go through the intervention modules and exercises again if they wish. If women do not wish to complete the intervention activities a second time, it is expected that 6 and 12 month visits will take approximately 15 min.

The actual time spent by women is recorded by the website analytics, and this will be used both for process evaluation and to inform cost-effectiveness.

### Randomisation and blinding

Participants are computer randomised automatically using simple randomisation, with no stratification, as the sample size is large enough that the groups should be balanced in terms of participant numbers and demographics. Once the participant has been randomly allocated to either the intervention or comparison study arm, they are sent a link to the appropriate website. They will not be informed as to whether the website they have been assigned to is the intervention or comparison site, although it is possible that they may guess which website they are using. All baseline measures are completed before women are exposed to the intervention or standard website. A list of participant usernames and passwords and study arm allocations is stored separately to participants’ names, ID numbers and contact details within the secure study database. All I-DECIDE investigators and members of the research team are blinded to the allocation of participants, until after collection and analysis of the 12 month data. Until this time, the two groups are referred to in tracking systems and other data collection as Group A and Group B (with the intervention group randomly allocated to either letter by the website developer/administrator). Follow up by the research assistant by phone for those who fail to log on and complete baseline, 6 month or 12 month measures is by an administrative assistant not connected with any of the data management or analyses.

### Retention

A number of strategies will be used in order to maximise retention, given that there will be limited contact with a research assistant:

- Use of friendly, warm communication strategies to enhance the feeling of support and safety;
- Regular emails to ‘touch base’ and remind participants about the study;
- Gift certificates to show appreciation for women taking the time to participate, at baseline and at 6 and 12 month follow-up (up to a maximum of $150 per participant across the study duration; e.g. $40 at baseline, $50 at 6 months and $60 at 12 months). Vouchers will be emailed to their nominated safe account with an accompanying email thanking them for their participation in a ‘Women’s Health Study’;
- Asking participants to confirm contact information between scheduled post-baseline surveys;
- Participants who have not logged in after two reminder emails will be phoned by an administrative assistant to encourage participation and establish reasons for non- participation

### Intervention arm (the I-DECIDE website)

The Psychosocial Readiness Model underpinning the I-DECIDE intervention, describes the interplay of factors that may motivate a woman experiencing domestic violence to engage in positive action for safety and healing [9]. It describes readiness as a continuum where there is a balance of internal and external factors determining how the woman moves from maintaining the status quo through to a desire for action. Rather than categorising women into a particular ‘stage’, the Psychosocial Readiness Model takes into account the fluid and changeable nature of women’s needs and wishes. It also acknowledges that women may define different things as ‘actions’, including health-seeking behaviours that do not have the end goal of leaving the abuser. Researchers increasingly support the use of this model in a domestic violence context [25].

Three internal factors are described as key to a woman’s readiness to change:

- *Awareness* is the woman’s recognition that what she is experiencing is abuse. A higher level of awareness/acceptance is usually linked to a greater desire for change.
- *Self-efficacy* is the woman’s belief that she is able to achieve difficult tasks, or cope with adversity.
- *Perceived support* describes the woman’s sense that she is supported by those in her environment. It may not reflect the level of actual support that is available.

In addition to the three internal factors, the model acknowledges the impact that external situational events can have on the change continuum (see Table 4). For example, gaining or losing employment, having access to an independent source of income, or a sudden health crisis. In light of this, the content of I-DECIDE has been designed to promote increased awareness and self-efficacy, while also aiming to improve levels of perceived support. Given the emphasis the theoretical model places on perceived support and awareness, the I-DECIDE intervention delivers its content in a therapeutic style. While it remains to be seen whether a web-based tool can provide the same level of support as a human being, the intervention provides tailored feedback and messaging to women at various stages so that they feel listened to, rather than being ‘just another user’. Additionally, it provides carefully-worded information about what a woman ‘might’ be experiencing, in order to raise her level of awareness around abusive behaviours. In order to increase self-efficacy, I-DECIDE encourages woman-led decision making and interactive elements to help her feel in charge of her own choices.

The intervention was informed by an ongoing process of consultation with women who have experienced violence or fear of a partner (focus groups with n = 23 women, and observational pilot sessions) and with community stakeholders (workshops with domestic violence organisations). These consultations led to various changes in language and tone, look and feel, and functionality.

The I-DECIDE website commences with three modules: healthy relationships, safety, and priorities. While a woman may choose which of the modules she wishes to begin with, she will need to complete the safety and priority modules to be able to receive the Action Plan strategies. The healthy relationship module is targeted at women who may not be ready to acknowledge that their relationship is abusive, but who want more information. The module outlines what a healthy relationship looks like, and asks a woman to indicate on a sliding scale how healthy she believes her own relationship to be, her current level of fear in the relationship, and her current level of safety. The safety module is primarily targeted at women who may be aware that their relationship is unhealthy, and who may be worried about their level of risk or danger. The module encourages further self-assessment and reflection of a woman’s relationship and level of safety and fear. It first directs her to complete the Composite Abuse Scale (CAS), a 30 item list of abusive behaviours which has been found in previous studies to encourage reflection that their relationship is abusive [26, 12]. This is followed by the Danger Assessment [16] to assess her level of risk. Women are provided with individualised feedback on both tools based on their results, which are automatically calculated by a computer algorithm. The priorities module provides women with the opportunity to weigh up different factors in a pairwise comparison. The priorities, which were developed by the IRIS team and refined through consultation with Australian stakeholders, are: my concern for safety, my health and wellbeing, having resources, and my feelings for my partner. A fifth option, children’s safety and wellbeing, is added if the woman has children. An algorithm calculates her top priority, which determines the strategies she will receive in the upcoming Action Plan.

At this point, if a woman has indicated that she is ambivalent to change or unaware that her relationship is abusive, she is directed to a motivational interviewing exercise [22]. This exercise encourages self-reflection through inputting free text around the pros and cons of the relationship with a partner or ex-partner. Once she has completed the motivational interviewing exercise, a woman is directed to the Action Plan. The Action Plan uses information provided throughout I-DECIDE to develop a tailored list of strategies. It takes into account a woman’s priorities, as well as her intentions for her relationship (stay, leave, already left). If a woman has scored as high risk in the safety module she is directed to an emergency safety plan with strategies such as locating a safe place to stay if the partner becomes abusive, taking copies of important documents, or using a code word to alert friends or family that she is in danger [27]. For women whose risk scores are lower, a list of five action plan strategies are automatically selected based on her top priority and whether she wishes to stay or leave the relationship. These strategies include resources and support contacts localised by State.

Lastly, women are directed to engage in a non-directive problem solving exercise. The non-directive problem solving exercise encourages a woman to choose one of the strategies from her action plan and work through any barriers she perceives are preventing her from carrying out that strategy. She is asked to input all the possible solutions that are available, and the advantages and disadvantages for each solution. In theory, this then leads to a clear preferred solution being identified which a woman has chosen herself and can pursue when she is ready [28].

### Comparison Arm

Women in the comparison arm are directed to an alternate version of the I-DECIDE website that represents the usual resources currently available through most domestic violence organisations. The comparison website includes the study measures as well as a standard “emergency safety plan” and links to national support service websites. It also includes static information on a healthy relationship, without any interactive elements.

### Safety procedures

Safety information is provided to all participants regarding the safe use of computers and the internet. Additionally, I-DECIDE is designed with a ‘quick escape’ bar that allows immediate exit and log out from I-DECIDE and returns the user to a generic website such as Google. A new browser window opens up with another generic website so that if a perpetrator forces a woman to click ‘Back’ on her browser it will not return to I-DECIDE. All automated emails sent to participants have the subject header “Women’s Health Study” and come from a specific email address (womens-health@unimelb.edu.au) that is separate to the regular project email address.

Should a woman contact a member of the research team because she is upset due to her participation in I-DECIDE, the researchers will refer to the study distress protocol in their response. The protocol includes exploring with the individual whether they have someone they can talk to who will understand and be supportive. The research team member will discuss with the participant where they might seek support, including the agencies detailed on the resource list provided to all participants. An independent Data Monitoring Committee (DMC) has been established in order to ensure that the trial is conducted appropriately. The DMC is scheduled to meet following major trial events and data collection milestones and at least annually. The DMC is composed of experts in randomised controlled trials and intimate partner violence. They will ensure that trial participants are protected, and will monitor the overall conduct of the trial.

### Outcomes

The primary outcomes, measures and hypotheses of the I-DECIDE trial are shown in Table 1 and the secondary outcomes in Table 2. Costs of managing and operating the website will be calculated from study records. Women’s time costs will be measured via recorded website analytics (time spent on website) and valued at the average Australian wage rate. Women’s use of services will be measured by responses to an adapted version of an instrument developed by Watson et al. [29] and valued using existing unit cost estimates. All costs will be presented in 2015 Australian dollars. Cost effectiveness will be assessed after 12 months as the additional cost per point change in self-efficacy and CESD-R. Harms and benefits of the study will also be explored using an adapted version of the COST Questionnaire [30].

### Data analysis

Characteristics of participants in each of the two study arms will be summarised using means and standard deviations or percentiles for continuous data, and frequencies and percentages for categorical data. Characteristics of participants in the two study arms will be compared at baseline to ensure that the randomisation was effective. Subsequent analyses will adjust for any baseline imbalances between groups strongly associated with the outcomes. Mixed effects linear regression will be used to compare scores between the two study groups on continuous outcomes [31]. Any count data which is skewed will be analysed using a Poisson model [32]. All regression models will adjust for baseline outcome measures and for any baseline imbalances strongly associated with the outcomes. Analyses will also take into account repeated measures over time (i.e. study group will be fitted as a fixed effect, and change over time within groups as random effects). A two-tailed alpha level of .05 will be used throughout analyses. Intention to treat and completed case analyses will be performed. Missing data will be assessed for patterns, including whether the data is missing completely at random (MCAR), missing at random (MAR), or missing not at random (MNAR) [33]. This will include analyses to determine whether particular participant characteristics or outcomes are associated with missing data, and whether any missing data patterns found are similar or different between intervention and comparison groups. Either multiple imputation or likelihood-based analysis will be used to account for missing data, depending on the nature of the missing data, and which assumptions are met [33].

### Process evaluation

A sub-sample (up to 25) of users will be recruited for the process evaluation. We will aim to recruit a maximum diversity sample: − women with different levels of fear; women who complete a module of the intervention vs. women who do not; women who revisit the site vs. women who only visit once; women who decide to leave vs. women who decide to stay; and women with children vs. women without. A research assistant will undertake interviews, following a topic guide including questions on how the participant first found the web-based intervention, their experience of using it, features they liked and disliked, recommendations for improvements or changes, and their perceptions of how using the intervention had impacted on their mental health and safety decision-making and planning processes. Particular attention will be paid to how women maintained safety and confidentiality, with a view to providing “top tips” to future users. All interviews will be tape-recorded and transcribed verbatim. The research assistant will keep additional field notes to capture contextual and non-verbal issues. Transcripts will be reviewed by a multi-disciplinary team to look for emerging themes and codes. Data will be coded by the research assistant, and themes that emerge will be discussed by the analysis group and explored in subsequent interviews.

## Discussion

In summary, there is a strong rationale for developing and testing online interventions to reach women and children who would not normally access traditional health and specialised services. This trial explores the effect of web-based relationship support and planning for safety and wellbeing, primarily on women’s self-efficacy and depressive symptoms. Well-designed theoretically informed randomised controlled trials that incorporate economic and process evaluations can provide us with the way forward to keep women and children safe and well. If successful, the I-DECIDE website could be easily incorporated into practice both within the community sector and health settings such as primary care. It also has the potential to function as a ‘first port of call’ for women who are unsure whether they need or wish to access any kind of formal support, allowing them to assess their relationship health before determining which pathway to take. The trial will also add to the knowledge base around internet-based trials, fully automated recruitment and retention processes. With an increasing demand for services and a community sector that is overworked the community needs novel ways of addressing the issue of domestic violence that are rigorously evaluated.

## Trial status

Currently recruiting.
